# Supplementary material for: Axonal dysfunction precedes axon degeneration in a mouse model of progressive parkinsonism
Source: bioRxiv. 2026 Oct 6:2026.09.29.755486. Preprint. [Version 1] doi: 10.64898/2026.09.29.755486 (PMC13691961; doi:10.64898/2026.09.29.755486)
Supplement: Supplementary file 1 [file NIHPP2026.09.29.755486V1-supplement-1.pdf]

bioRxiv preprint doi: https://doi.org/10.64898/2026.09.29.755486; this version posted October 6, 2026. The copyright holder for this preprint (which was not certified by peer review) is the author/funder. This article is a US Government work. It is not subject to copyright under 17 USC 105 and is also made available for use under a CC0 license.

## SUPPLEMENTAL MATERIALS

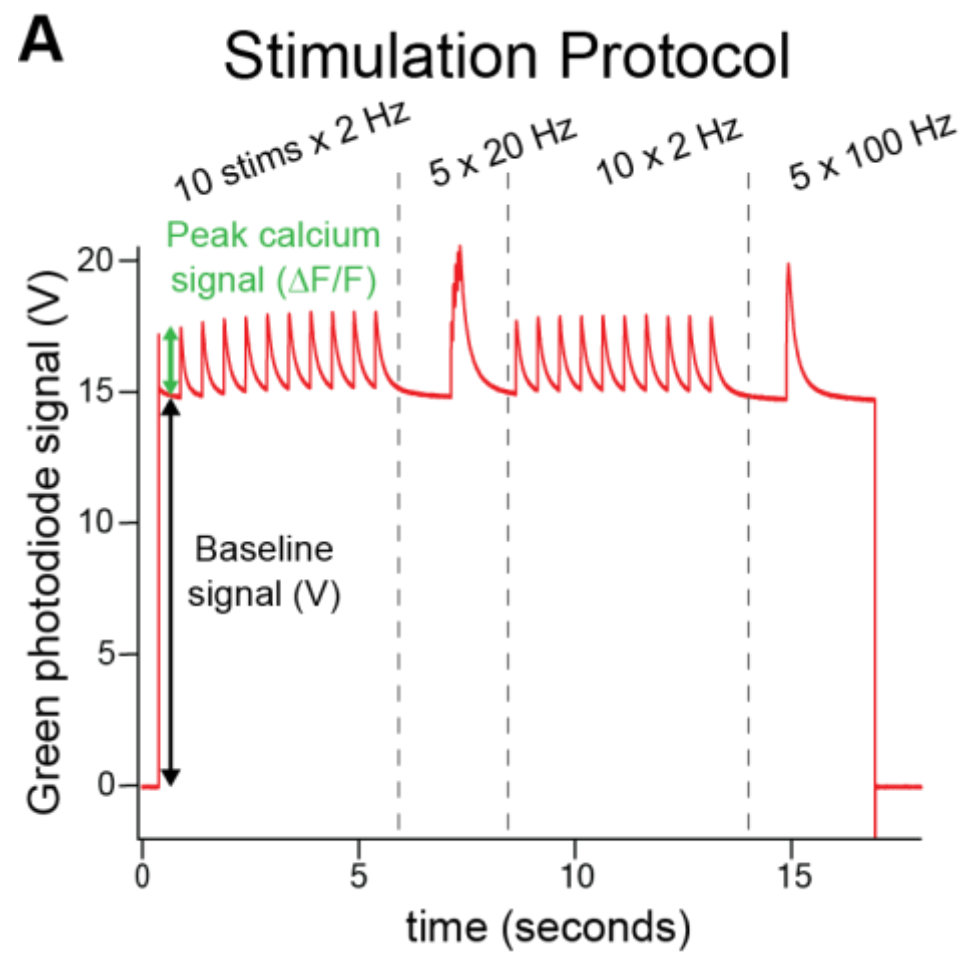


**Supplemental Figure 1: Nigrostriatal axons from MitoPark mice show an age-dependent reduction in baseline calcium signals**

*A.* Stimulation protocol used to measured evoked calcium influx in nigrostriatal axons. Baseline calcium was measured as the photodiode signal, in volts, after the photodiode turned on but before activity was stimulated.

bioRxiv preprint doi: https://doi.org/10.64898/2026.09.29.755486; this version posted October 6, 2026. The copyright holder for this preprint (which was not certified by peer review) is the author/funder. This article is a US Government work. It is not subject to copyright under 17 USC 105 and is also made available for use under a CC0 license.

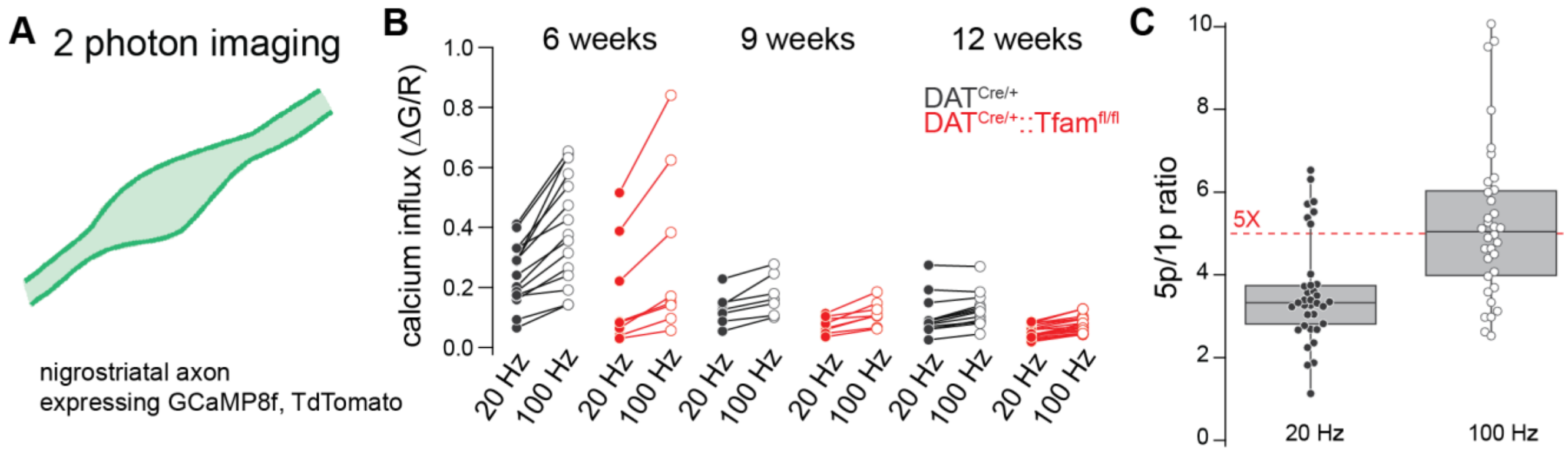


**Supplemental Figure 2: Calcium influx does not saturate at 20 Hz in nigrostriatal axons**

- ***A.*** Schematic showing experimental approach. Calcium influx was measured for single axons using two-photon laser microscopy. Axons were stimulated with 5 pulses at 20 Hz and 5 pulses at 100 Hz.
- ***B.*** Summary of calcium influx measured in single axons in response to trains of 20 and 100 Hz stimulation. Lines connect signals recorded from the same axon. In all ages and genotypes, 100 Hz stimulation leads to greater calcium influx than 20 Hz stimulation ($p < 0.05$, Wilcoxon matched-pairs signed rank tests).
- ***C.*** The ratio of peak calcium influx at the fifth pulse relative to the calcium associated with the first pulse shows that AP-associated calcium influx in single axons is largely within the linear range of the jGCaMP8f sensor.

bioRxiv preprint doi: https://doi.org/10.64898/2026.09.29.755486; this version posted October 6, 2026. The copyright holder for this preprint (which was not certified by peer review) is the author/funder. This article is a US Government work. It is not subject to copyright under 17 USC 105 and is also made available for use under a CC0 license.

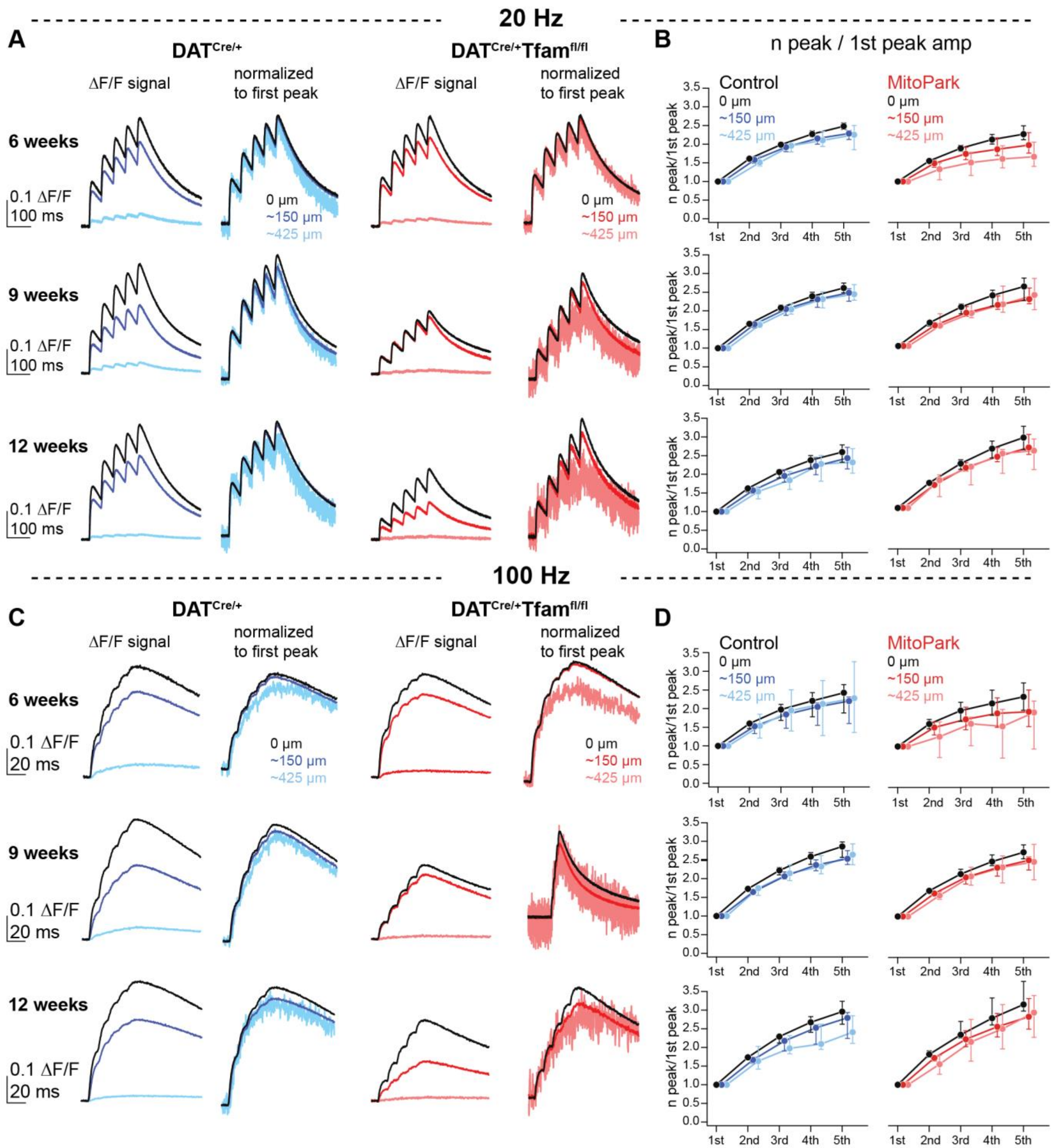


**Supplemental Figure 3: Calcium influx decreases with distance for high-frequency bursts in nigrostriatal axons**

- ***A.*** Left column: Representative ΔF/F signals for bursts (5 pulses) at 20 Hz in $DAT^{Cre/+}$ (blues) and $DAT^{Cre/+}$::$Tfam^{fl/fl}$ (reds). Right column: Same traces shown in left column normalized to the first of five pulses.
- ***B.*** Relationship between peak number and normalized calcium influx for both genotypes at three distances, 0 µm from stimulating electrode (black), 150 µm (blue, red), and 425 µm (light blue, light red). Data are shown as median and interquartile range.
- ***C.*** Same as panel A but for 5 pulses of 100 Hz stimulation.

bioRxiv preprint doi: https://doi.org/10.64898/2026.09.29.755486; this version posted October 6, 2026. The copyright holder for this preprint (which was not certified by peer review) is the author/funder. This article is a US Government work. It is not subject to copyright under 17 USC 105 and is also made available for use under a CC0 license.

***D.*** Peak number and normalized calcium influx for 100 Hz stimulation at different distances from the stimulating electrode.

bioRxiv preprint doi: https://doi.org/10.64898/2026.09.29.755486; this version posted October 6, 2026. The copyright holder for this preprint (which was not certified by peer review) is the author/funder. This article is a US Government work. It is not subject to copyright under 17 USC 105 and is also made available for use under a CC0 license.

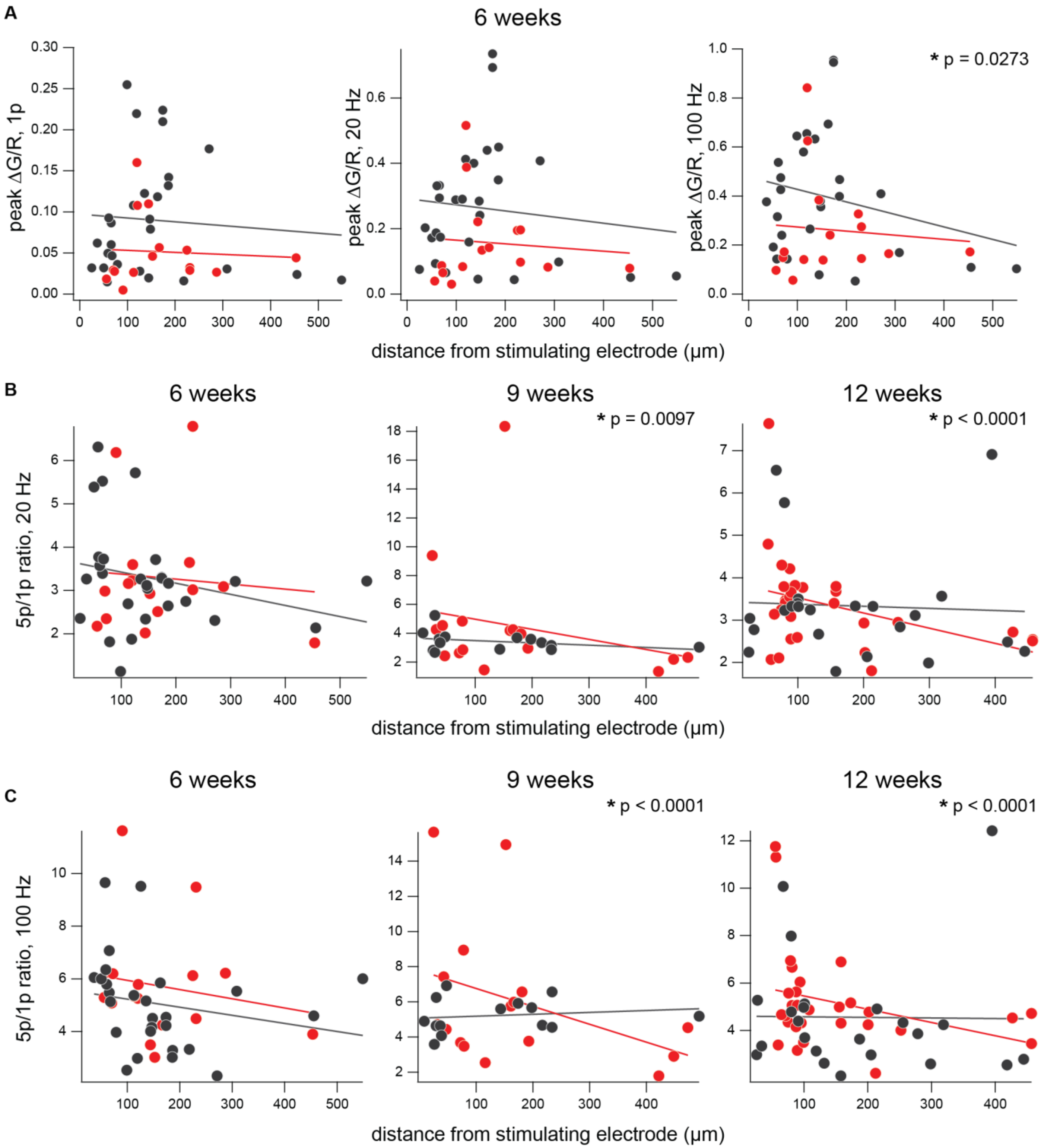


**Supplemental Figure 4: Calcium influx decreases in a frequency- and distance-dependent manner in MitoPark axons**

- ***A.*** Relationship between peak calcium influx and distance from the stimulating electrode for single pulses, 20 Hz, and 100 Hz stimulation in 6-week animals of both genotypes. The line of best fit was calculated for each genotype at each stimulation frequency.
- ***B.*** The relationship between 5p/1p ratio and distance for 20 Hz stimulation at each timepoint. At 9 and 12 weeks, MitoPark axons showed a distance-dependent decrease in this ratio.

bioRxiv preprint doi: https://doi.org/10.64898/2026.09.29.755486; this version posted October 6, 2026. The copyright holder for this preprint (which was not certified by peer review) is the author/funder. This article is a US Government work. It is not subject to copyright under 17 USC 105 and is also made available for use under a CC0 license.

*C.* As in B, but for 100 Hz stimulation. As above, 9- and 12-week MitoPark axons showed a negative correlation between 5p/1p ratio and distance, while control axons did not.

bioRxiv preprint doi: https://doi.org/10.64898/2026.09.29.755486; this version posted October 6, 2026. The copyright holder for this preprint (which was not certified by peer review) is the author/funder. This article is a US Government work. It is not subject to copyright under 17 USC 105 and is also made available for use under a CC0 license.

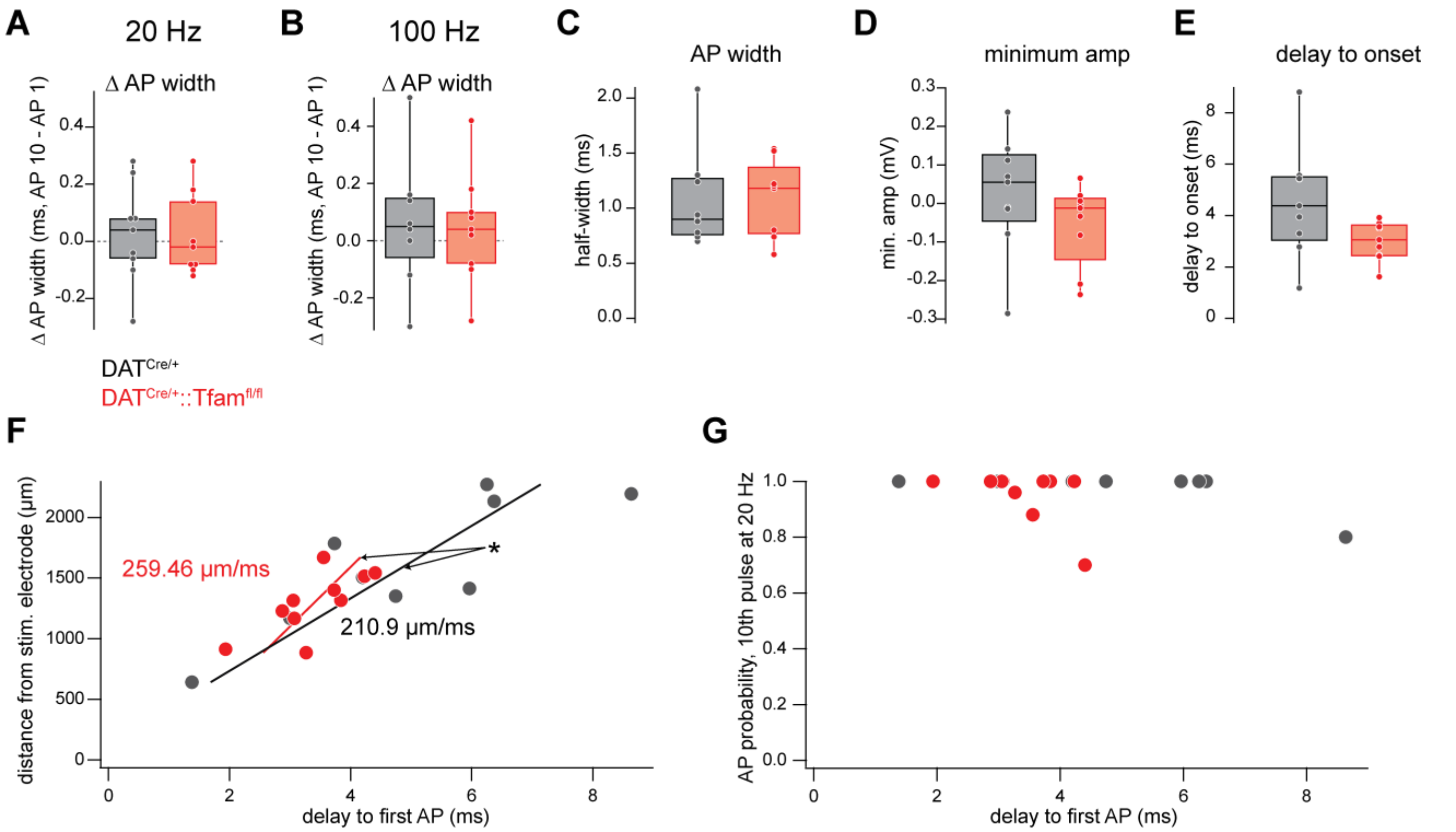


**Supplemental Figure 5: Cell-attached AP properties do not differ between genotype at 9 weeks**

- ***A.*** AP width, measured as the time between cell-attached AP maximum amplitude to minimum amplitude trough, did not change with 20 Hz stimulation.
- ***B.*** Both MitoPark and control axons show no change in AP width with 100 Hz firing.
- ***C.*** Overall, there was no difference in cell-attached AP width between MitoPark and control axons.
- ***D.*** The minimum amplitude of the cell-attached AP waveform across genotypes at 9 weeks.
- ***E.*** Delay to AP onset, measured from stimulus offset to AP maximum amplitude.
- ***F.*** The relationship between delay to AP onset and distance from the stimulation electrode showed a positive correlation in both genotypes.
- ***G.*** The relationship between delay to AP onset and AP probability for the 10$^{th}$ AP in a 20 Hz burst for both genotypes.
